# Maternal Immune Activation Alters Adult Behavior, Gut Microbiome and Juvenile Brain Oscillations in Ferrets

**DOI:** 10.1523/ENEURO.0313-18.2018

**Published:** 2018-10-31

**Authors:** Yuhui Li, Supritha R. Dugyala, Travis S. Ptacek, John H. Gilmore, Flavio Frohlich

**Affiliations:** 1 Department of Psychiatry, University of North Carolina at Chapel Hill, Chapel Hill, NC 27599; 2 Neuroscience Center, University of North Carolina at Chapel Hill, Chapel Hill, NC 27599; 3 Neuroscience Center Bioinformatics Core, University of North Carolina at Chapel Hill, Chapel Hill, NC 27599; 4 Department of Neurology, University of North Carolina at Chapel Hill, Chapel Hill, NC 27599; 5 Department of Cell Biology and Physiology, University of North Carolina at Chapel Hill, Chapel Hill, NC 27599; 6 Department of Biomedical Engineering, University of North Carolina at Chapel Hill, Chapel Hill, NC 27599; 7 Carolina Center for Neurostimulation, University of North Carolina at Chapel Hill, Chapel Hill, NC 27599

**Keywords:** behavior, developmental disorders, ferrets, maternal immune activation, microbiome, oscillations

## Abstract

Maternal immune activation (MIA) has been identified as a causal factor in psychiatric disorders by epidemiological studies in humans and mechanistic studies in rodent models. Addressing this gap in species between mice and human will accelerate the understanding of the role of MIA in the etiology of psychiatric disorders. Here, we provide the first study of MIA in the ferret (*Mustela putorius furo*), an animal model with a rich history of developmental investigations due to the similarities in developmental programs and cortical organization with primates. We found that after MIA by injection of PolyIC in the pregnant mother animal, the adult offspring exhibited reduced social behavior, less eye contact with humans, decreased recognition memory, a sex-specific increase in amphetamine-induced hyperlocomotion, and altered gut microbiome. We also studied the neurophysiological properties of the MIA ferrets in development by *in-vivo* recordings of the local field potential (LFP) from visual cortex in five- to six-week-old animals, and found that the spontaneous and sensory-evoked LFP had decreased power, especially in the gamma frequency band. Overall, our results provide the first evidence for the detrimental effect of MIA in ferrets and support the use of the ferret as an intermediate model species for the study of disorders with neurodevelopmental origin.

## Significance Statement

Maternal immune activation (MIA) has been adopted in the rodent model to study neurodevelopmental disorders such as schizophrenia. However, neurodevelopmental programs differ quite substantially between mice and humans. The ferret has a rich history for the study of neurodevelopment due to its unique advantages that combine short gestation time with the emergence of sophisticated cortical organization during development. The present study found that MIA leads to a range of behavioral abnormalities as well as altered gut microbiome in adult ferrets. Notably, we observed impaired brain oscillations in these animals in early development. Our results lay the foundation for the translational study of neurodevelopmental disorders in ferrets.

## Introduction

Environmental factors such as maternal infection during early development contribute to the etiology of many psychiatric disorders such as autism spectrum disorder and schizophrenia (Grabrucker, 2013; Murray et al., 2017). Epidemiological studies show that the risk of psychiatric disease increases in the offspring after maternal illness during pregnancy (Mednick et al., 1988; Brown et al., 2004; Byrne et al., 2007; Atladóttir et al., 2010; Abdallah et al., 2012; Zerbo et al., 2013). The elevated risk is associated with the activation of the maternal immune system (Jarskog et al., 1997; Gilmore and Jarskog, 1997; Urakubo et al., 2001; Brown et al., 2004; Gilmore et al., 2004, 2005; Smith et al., 2007; Abdallah et al., 2012; Choi et al., 2016). These observations sparked researchs of maternal immune activation (MIA) in animals to study the effect of such a prenatal perturbation on brain development in the context of neurodevelopmental disorders. These studies showed a broad range of behavioral, neuroanatomical, and neurochemical changes as a consequence of MIA and revealed the underlying mechanisms, such as changes of the maternal and fetal cytokines, altered stress pathways, and deficits of certain types of interneurons (Urakubo et al., 2001; Gilmore et al., 2005; Boksa, 2010; Patterson, 2011; Piontkewitz et al., 2012; Kneeland and Fatemi, 2013; Knuesel et al., 2014; Meyer, 2014; Reisinger et al., 2015; Estes and McAllister, 2016).

Most of the MIA animal studies, however, focus on rodents. To amplify translational relevance, it is necessary to verify whether the results can be generalized across species. Recent studies confirmed that MIA also elicits some behavioral deficits (Willette et al., 2011; Bauman et al., 2014; Machado et al., 2015) and anatomic abnormalities (Short et al., 2010; Weir et al., 2015) in juvenile rhesus monkeys. However, the specific phenotypes found in the monkeys were distinct from those found in rodents, suggesting species differences of the effects of MIA. Furthermore, while MIA in mice generally results in large effects, prenatal infections in humans do not have the same consistent and pronounced effects. Many factors, like the genetic background and the vulnerability to environmental insults, may contribute to this difference (Meyer, 2014), thus emphasizing the importance of the model to be verified in species with a more heterogeneous genetic background as well as a longer development period.

The domestic ferret has been widely used in studies of neurodevelopment, brain diseases and immunology. Ferrets are extremely altricial animals. Their neurodevelopment in the first four postnatal weeks are equivalent to the last trimester of pregnancy in humans (Clancy et al., 2001; Medina et al., 2005). This is the main reason why the ferret is used to study the development of visual system (Chapman and Stryker, 1993; Li et al., 2008; Smith et al., 2015). Furthermore, the ferret cortex is smooth at birth but starts to fold (gyrification) from postnatal day (P)10 (Borrell and Reillo, 2012; Sawada and Watanabe, 2012; Knutsen et al., 2013), making ferrets particularly suitable for studies of brain trauma and other insults during development (Medina et al., 2005; Empie et al., 2015; Trindade et al., 2016; Schwerin et al., 2017). With an immune system similar to human, ferrets are also widely used to study the immune response to virial infections (Belser et al., 2011, 2016). The ferret has a well-defined prefrontal cortex (Duque and McCormick, 2010), which makes it suitable to study cognition. Ferret are social animals and have shown promise for the study of socio-cognitive functions (Hernádi et al., 2012). Finally, ferrets have a moderate gestation time (∼41 d) and relative large litter size (8–18; Ball, 2006) compared to monkeys.

Here, we studied the effect of MIA on the adult ferret offspring. We adopted behavioral tests that have been used in rodents to model the behavior construct of several neurodevelopmental disorders (Meyer, 2014; Estes and McAllister, 2016). We also used some ferret-related tests to measure changes in cognitive and social behaviors (Poole, 1972; Hernádi et al., 2012). To further investigate the effect of MIA and its possible underlying mechanisms, we also performed preliminary studies of the gut microbiome in adults and brain oscillatory activity in the juvenile animal.

## Materials and Methods

### Animals

Twelve pregnant ferrets (*Mustela putorius furo*) were acquired at the gestational age of day 21–24 (G21–G24) from Marshall BioResources Inc. and housed individually. At G30, they were randomly assigned to receive either 10 mg/kg PolyIC (polyinosinic:polycytidylic acid, potassium salt; Sigma-Aldrich; dissolved 10 mg/ml in PBS) or 1 ml/kg PBS by intraperitoneal administration under anesthesia introduced by 4–5% isoflurane then sustained by 1–2%. To confirm the activation of the immune system, rectal temperature was measured immediately before and 3 h after the injection. Blood samples (0.5 ml) were drawn from the jugular vein at the same time points. Serum cytokine levels (IL-2, IL-6, and TNFα) were determined by the University of North Carolina at Chapel Hill Animal Clinical Chemistry and Gene Expression Laboratory using multiplexed biomarker immunoassays (Luminex MAGPIX system, Luminex Inc., using the canine cytokine kit). The detection sensitivities (mean + 2 SD) were 3.5 pg/ml for IL-2, 3.7 pg/ml for IL-6, and 6.1 pg/ml for TNFα. Ferret offspring (kits) were born at ∼G40–G41. The kits were kept with their mother until weaning at P42, when they were separated into cages by sex. The males were single-housed at approximately three to four months old when they became progressively aggressive and caused lesions when fighting each other (Ball, 2006). To minimize the effect of single-housing of the males on their behavior, their cages were close to each other to allow visual, auditory, and olfactory interactions, and enrichment was provided. The female offspring were group-housed with three to four animals per cage. The offspring of three pregnant ferrets receiving PolyIC were used for the electrophysiology study. Another nine litters (four receiving maternal PolyIC injections and five receiving PBS injections) were kept until the age of six months for the investigation of behavior and microbiome. We excluded one litter of kits since the jill had very high level of cytokines (>2000 pg/ml for all factors) at baseline before the PolyIC injection. As a result, a total twelve kits (four males and eight females) were used in the electrophysiology study, and a total of forty-five kits were used in the behavioral study (PolyIC: 10 males and 15 females, PBS: 11 males and nine females; for detailed litter information, see Table 1). The pregnant ferrets and their kits were housed in a 16/8 h light/dark cycle throughout the pregnancy and nursing periods to ensure the same breeding season cycle as maintained by the supplier. After weaning the kits were turned to 12/12 h light/dark cycle. All procedures were approved by the University of North Carolina at Chapel Hill–Chapel Hill Institutional Animal Care and Use Committee (UNC-CH IACUC) and in compliance with the guidelines set forth by the NIH (NIH Publications No. 8023, revised 1978) and United States Department of Agriculture.

**Table 1.** Information of the animals used in this study

			# of offspring	
Litter #	Treatment	Study	Male	Female
1	PolyIC	Electrophysiology	1	3
2	PolyIC	Electrophysiology	0	4
3	PolyIC	Electrophysiology	3	1
4	PolyIC	Behavior	1	3
5	PolyIC	Behavior	1	2
6	PolyIC	Behavior	3	7
7	PolyIC	Behavior	5	3
8	PBS	Behavior	2	2
9	PBS	Behavior	2	2
10	PBS	Behavior	4	3
11	PBS	Behavior	3	2
12^a^	PBS	Excluded	4	1

a The litter was excluded from further analysis because of high serum cytokine levels at baseline before injection.

### Experimental design of the behavioral tests

When the offspring reached the age of six months, four behavioral tests were performed in the following order: open field exploration, novel object recognition, social interaction, amphetamine-induced hyperlocomotion. Different tests were conducted on separate days. We completed the investigation of the females after finishing the above tests because female ferrets usually begin estrus at the age of seven months. The estrus requires immediate medical actions (spay or gonadorelin administration) to prevent serious health consequences, but any of these treatments may inevitably complicate the interpretation of the behavioral results. Additional four behavioral tests were conducted on the remaining males: MK-801-induced hyperlocomotion, engagement with salient stimulus, eye contact tolerance, and adaptation to repeatedly auditory stimuli. To minimize the effects of dopaminergic sensitization by amphetamine or MK-801 on subsequent tests, at least one month elapsed before the next test was performed. We had also tried the auditory startle and the pre-pulse inhibition test; however pilot results indicated that ferrets do not exhibit any easily observable responses on the startle sound (50-ms pulse of 120 dB SPL). All tests were performed in a well-illuminated 1.5 × 1.5 m^2^ arena unless specified. The arena was cleaned with 70% ethanol between tests on different animals. The tests were conducted by experimenters blind to the group membership of the animals.

### Open field exploration

The animal was placed in the arena and allowed to freely explore for 15 min. The activity within the arena was captured by a top-mounted camera (Microsoft LifeCam Cinema 720p HD Webcam, 30 Hz frame rate).

### Novel objection recognition

The animal was first introduced into the arena for 3 min to acclimate. Then the learning phase began: the animal was temporally removed from the arena, and two identical objects were placed into two opposite corners of the arena and 15 cm away from the walls. Then the animal was placed back to the arena for 5 min. The recall phase started two and half hours after the learning phase. One of the two objects was replaced with a novel object. The animal was placed in the cage for 5 min and its interaction with the objects was video recorded. The novel object recognition is characterized as the time the animal spends interacting with the novel object minus the time interacting with the familiar one. Two types of objects (black dumbbell and green kettlebell) were used in the test. The identity of the novel object and the novel object location were randomized and balanced across animals of the two treatment groups. All objects were cleaned with 70% ethanol between testing phases.

### Social interactions

In the acclimating phase, two identical cages were placed in two sides of the arena and 15 cm away from the walls. The cages were 49 × 33 × 26 cm^3^ (L × W × H) and had “barred windows” at all four sides. The animal was placed in the arena for 10 min before temporally removed from the arena. Then a stranger ferret (not used in the study) with the same sex was placed randomly in one of the cages. The test animal was placed back to the arena for 10 min (sociability test phase). The sociability is characterized as the time the test animal interacts with the cage containing the stranger animal minus the time the test animal interacts with the empty cage. Then, another stranger animal was placed in the other cage. And the test animal was placed to the arena for 10 min (social preference test phase). The social preference is characterized as the time the test animal interacts with the cage containing the new animal minus the time it interacts with the cage containing the animal that was previously introduced. The order of the stranger animals put in the test were randomized and balanced across animals of the two treatment groups. For randomly half of the animals tested, we switched the locations of the cage that contained the familiar animal or the stranger animal in the social preference test. The purpose of this manipulation was to disassociate the preference to a new animal versus the preference to an animal in a new location. However, no obvious preference to new animal locations was observed so we grouped the conditions of cage exchanging in further analysis. All cages were cleaned with 70% ethanol between tests.

### Amphetamine-induced hyperlocomotion

After acclimation in the arena, the animal was injected intraperitoneally with 1 ml/kg saline before being placed back to the arena for 1 h. Next, the animal was injected intraperitoneally with 1 mg/kg D-amphetamine (hemisulfate salt, Sigma-Aldrich; dissolved 1 mg/ml in saline) and was put back into the arena for 2 h. The activity of the animal was video recorded.

### MK-801-induced hyperlocomotion

The configuration of this assay was similar to the amphetamine-induced hyperlocomotion test expect that we injected the animals intraperitoneally with 0.15 mg/kg MK-801 [(+)-MK-801 hydrogen maleate, Sigma-Aldrich; dissolved 0.15 mg/ml in saline].

### Engagement with salient stimulus

This naturalistic attention test was conducted in the home cage in the animal facility, which is a cage with transparent walls all around. One experimenter stood in front of the cage. The test started once the animal attended and approached the experimenter. The experimenter made sounds by rattling a chain of keys or by a jar containing colorful metal pins to attract attention. If the animal approached and explored the sound source for a few seconds, the experimenter would move the keys or jar to a new position around the cage to see whether the animal could keep attending and following the sound source. If the animal failed to attend and follow the relocated sound source after a few seconds, the experimenter put it back closer to make the animal engaged again. The test lasted for 2 min, and the behavior of the animal was videotaped for subsequent scoring.

### Eye-contact tolerance

The eye-contact test was also conducted in the animal facility. One experimenter took the animal out from the housing cage and held the animal at the level of the experimenter’s face and ∼25 cm away. The experimenter tried to attract attention by talking and making facial expression. The test lasted for 30 s, and the behavior of the animal was videotape by a head-mounted camera for subsequent scoring.

### Adaptation to repeated noises

The test was adopted from a previous study (Poole, 1972) and was conducted in an 80 × 80 cm^2^, sound-insulated and well-illuminated box. Enrichments including shoe covers, papers, and juggle balls were placed inside to keep the animals active. The animal was acclimated in the box for 45 min. Then a 5-s clip of paper crackling sounds was played once per minute for 40 min. The intersound interval was set to vary from 45 to 75 s to reduce expectation. The sound was played by a speaker mounted on one side of the wall of the box. The activity of the animal within the box was captured by a top-mounted camera.

### Microbiome sample collection and sequencing

Stool samples were collected from twenty randomly selected ferrets used above (balanced for sex and group, five males and five females from the PolyIC group and five males and five females from the PBS group) when they became adult (six months) and before the start of any behavioral tests. The samples were collected in the morning after moving the animals to freshly disinfected cages. The inner part of the stool without any mucosae was separated immediately after defecation, frozen in dry ice-ethanol bath, and stored in -80°C. The 16S rDNA amplicon sequencing was performed at the University of North Carolina Microbiome Core Facility (Chapel Hill, NC). In brief, DNA was extracted from the stool contents by MagMAX Total Nucleic Acid Isolation kit (Thermo Fisher Scientific); 12.5 ng of total DNA was amplified using primers consisting of the locus-specific sequences targeting the V3-V4 region of the bacterial 16S rDNA (Edwards et al., 1989; Fierer et al., 2008; Caporaso et al., 2011). Primer sequences contained overhang adapters appended to the 5’ end of each primer for compatibility with Illumina sequencing platform. The complete sequences of the primers were: forward: 5’ TCGTCGGCAGCGTCAGATGTGTATAAGAGACAG GTGCCAGCMGCCGCGGTAA 3’; and rewind: 5’ GTCTCGTGGGCTCGGAGATGTGTATAAGAGACAGGGACTACHVGGGTWTCTAAT 3’.

Master mixes contained 12.5 ng of total DNA, 0.2 µM of each primer and 2x KAPA HiFi HotStart ReadyMix (KAPA Biosystems). The thermal profile for the amplification of each sample had an initial denaturing step at 95°C for 3 min, followed by a cycling of denaturing of 95°C for 30 s, annealing at 55°C for 30 s, and a 30 s extension at 72°C (25 cycles), a 5 min extension at 72°C and a final hold at 4°C. Each 16S amplicon was purified using the AMPure XP reagent (Beckman Coulter). In the next step, each sample was amplified using a limited cycle PCR program, adding Illumina sequencing adapters and dual‐index barcodes (index 1(i7) and index 2(i5); Illumina) to the amplicon target. The thermal profile for the amplification of each sample had an initial denaturing step at 95°C for 3 min, followed by a denaturing cycle of 95°C for 30 s, annealing at 55°C for 30 s and a 30 s extension at 72°C (eight cycles), a 5-min extension at 72°C, and a final hold at 4°C. The final libraries were again purified using the AMPure XP reagent (Beckman Coulter), quantified, and normalized before pooling. The DNA library pool was then denatured with NaOH, diluted with hybridization buffer and heat denatured before loading on the MiSeq reagent cartridge (Illumina) and on the MiSeq instrument (Illumina). Automated cluster generation and paired–end sequencing with dual reads were performed according to the manufacturer’s instructions.

### *In vivo* electrophysiological recording

Multi-electrodes recording were performed at P22–P50 following the same procedure as in our previous study (Li et al., 2017). Briefly, the surgery for implanting the electrode arrays was performed one to three d before the recording. Anesthesia was induced with 4–5% isoflurane then maintained by 1.5–3% isoflurane in 100% medical grade oxygen. Lidocaine (2%) was used for topical analgesia and furosemide (5%, 0.04 ml/kg) was used to prevent cerebral edema. The electrocardiogram, breathing rate, and body temperature were monitored throughout the surgery to maintain deep general anesthesia. Body temperature was maintained within 36–38°C by hot-snap pads and a water heating blanket. Animals were fixed in a stereotaxic frame (David Kopf Instruments). The craniotomy was made over visual cortex located 1–3 mm anterior from lambda and 6–9 mm lateral from midline. The dura and pia were removed. A 2 × 8 electrode array (Innovative Neurophysiology; 1-MΩ impedance, 200-μm spacing, 0.5-mm shorter low-impedance reference electrode) was lowered down into the cortex until spikes or local field potential (LFP) signals were recorded. The array was then fixed to the skull by dental cement and bone screws. After surgery, the kit was returned to the litter. The body weight was measured twice a day for the following days to ensure proper recovery. Acetaminophen (Children’s Tylenol, 16 mg/kg) was administrated orally twice per day for at least 3 d after surgery for pain alleviation.

Recordings took place in a light-insulated ferret cage with bedding. Spontaneous activity was recorded when the animal freely moved in the cage for 10–15 min. Then visual-evoked activity was recorded when visual stimuli were displayed by four computer-controlled LED lights positioned in each corner of the cage. Each stimulus was 500 ms in duration, and it was repeated 100–200 times. Each recording session lasted less than 1 h. The neural signal recorded from the electrode arrays were amplified and digitized by a light-weight head-stage (Intan; RHD2132, 20-kHz sampling rate). The signal was transmitted to an electrophysiology acquisition system (Intan, RHD2000) and then to a computer for *post hoc* analysis. An infrared sensitive camera simultaneously recorded the behavior of the animal. The video and the neural recording data were synchronized by a computer-controlled infrared LED light. Immediately after recordings, animals were euthanized with an overdose of sodium pentobarbital and perfused with 0.1 M PBS followed by 4% formaldehyde. The brain was extracted and sliced to reconstruct the electrode tracks.

### Data analysis of the behavioral data

The videos recorded in the behavior test were processed by EthoVision software (Noldus). Locomotion and location data were extracted for open field and the amphetamine and MK-801 induced hyperlocomotion tests. To study the preference of the animal to stay in the center versus periphery in the open field, the center region was defined as the center 70 × 70 cm^2^ area, which was 40 cm away from the wall, approximating the body length of an adult male ferret. We used the BORIS software (Friard and Gamba, 2016) to manually log behavioral events in the following tasks: novel object recognition, social interaction, engagement with salient stimuli, eye-contact tolerance, and responses to repeated noises. BORIS enables frame-by-frame analysis of a video and labeling the start and end time of an event. For the videos of the novel object recognition task, we logged the timing when the animal moved their nose to actively explore either of the objects. For the social interaction videos, we logged the timing when the animal explored either of the cages. For logging the videos of engagement with salient stimuli, we characterized the attention/reaction to the stimuli using the following scheme: 1 for when an animal paid no attention to the stimuli and engaged in other behaviors, 2 for when an animal attended the stimuli by moving its head, 3 for when an animal moved to follow the stimuli, 4 for when an animal was fully engaged with the stimuli and tried to scratch it. For the eye-contact tolerance test, we detected the eye contacts which were defined as when the animals orientated and maintained their gaze toward the head-held camera for at least 300-ms. For the auditory attention test, we characterized the behavioral responses to each repetition of sound as “attention response,” “partial response,” or “no response” using the criteria from a previous study (Poole, 1972). Attention response was defined as when the animals raised their neck, held their head at 90° to the body and pricked their ears. Partial response was defined as when the animal showed partial but not full attention responses. The logging of the videos was performed by an experimenter blind to the group identity of the animal. To confirm the logging result, another experimenter independently logged the videos and we assured that the two logs to individual video files overlapped at least 90%.

### Data analysis of the microbiome data

For analyzing the microbiome data, multiplexed paired-end fastq files were produced from the sequencing results of the Illumina MiSeq using the Illumina software configureBclToFastq. The paired-end fastqs were joined into a single multiplexed, single-end fastq using the software tool fastq-join. Demultiplexing and quality filtering was performed on the joined results. Quality analysis reports were produced using the FastQC software. Bioinformatics analysis of bacterial 16S amplicon sequencing data were conducted using the Quantitative Insights Into Microbial Ecology (QIIME) software (Caporaso et al., 2010). OTU picking was performed on the quality filtered results using pick_de_novo_otus.py. Chimeric sequences were detected and removed using ChimeraSlayer. α Diversity and β diversity analysis were performed on the data set using the QIIME routines: alpha_rarefaction.py and beta_diversity_through_plots.py (Lozupone et al., 2006), respectively. Summary reports of taxonomic assignment by sample and all categories were produced using QIIME summarize_taxa_through_plots.py and summarize_otu_by_cat.py. To test differences at the population level (β diversity), samples were clustered using weighted and un-weighted unifrac clustering (Lozupone and Knight, 2005) and grouped by sex and treatment. Differences in β diversity by group were tested using the PERMANOVA test (*p* < 0.05 considered significant) as implemented by QIIME’s compare_categories.py. To test differences at the taxa level, we used the Kruskal–Wallis test (FDR corrected *p* < 0.05 considered significant) as implemented by QIIME’s group_significance.py. Taxa were tested at the phylum, class, order, family, and genus levels.

### Data analysis of the electrophysiology data

The electrophysiology results were compared between the MIA offspring from three litters and the results from a previous study (Li et al., 2017) to minimize the number of animals used. The lack of direct comparison of two groups of animals from the same study is a limitation of this pilot work. Data were analyzed by custom-written scripts in MATLAB (MathWorks). LFP and multi-unit activity (MUA) signals were extracted by applying a 300-Hz low-pass filter and a 300-Hz high-pass filter, respectively, to the raw data (60-Hz line-noise removed). The LFP spectrogram was computed by convolving LFP signals with a family of Morlet wavelets (0.5–120 Hz in 0.5-Hz steps). The mean power spectrum was estimated by averaging the square of the absolute value of the convolved signal across the time of interest. To account for the power law scaling of the LFP power spectrum, the power spectra were 1/f normalized by multiplying each data point with its frequency.

Spikes were extracted using a threshold of minus-five-times the SD of the high-pass filtered signal. Visual response latency was estimated using a previous method (Maunsell and Gibson, 1992). Visual response variance across trials was characterized by coefficient of variance (CV), defined as the SD of the firing rate in 0–500 ms after stimulus onset divided by the mean firing rate during the same time period.

### Statistical analysis

The results are represented as mean ± SD unless specified. Statistics were calculated by MATLAB function ttest2 for unpaired Student’s *t* test, anovan for unbalanced ANOVA test, and multcompare for *post hoc* multiple comparison test with the Tukey–Kramer method.

## Results

To determine whether MIA perturbs developmental outcomes in the ferret, we administered pregnant ferrets with either 10 mg/kg PolyIC or PBS on G30 and studied the progeny with a combination of methods that included a comprehensive battery of behavioral tasks, which were assembled to include the typical assays used in rodent MIA studies and tasks that have been previously used in ferrets. In addition, we performed pilot studies of electrophysiological recordings and sequencing of the gut microbiome.

### Maternal immune response, survival rate of the kits, and birth weight

We confirmed that PolyIC triggered a maternal immune response by measuring the body temperature and maternal serum cytokine IL-2, IL-6, and TNFα 3 h after the injection. Both the body temperature and cytokine levels were significantly higher in the jills that received PolyIC than in those that received PBS (body temperature after injection: PolyIC = 39.83 ± 0.96°C, *n* = 7; PBS = 37.90 ± 0.22°C, *n* = 4, unpaired *t* test, *t*_(9)_ = 3.41, *p* = 0.008; for result of serum cytokine changes, see Table 2).

**Table 2.** Cytokine level before and 3 h after PolyIC or PBS injection

	Cytokine concentration (pg/ml)			
	PBS (*n* = 4)		PolyIC (*n* = 7)	
	Before	After	Before	After
IL-2	6.6 ± 7.6	5.6 ± 6.4	5.6 ± 8.4	111.9 ± 166.0^#^
IL-6	9.2 ± 8.0	7.8 ± 6.9	15.4 ± 13.6	119.1 ± 124.4^*^
TNFα	6.1 ± 7.0	4.9 ± 5.8	8.9 ± 5.4	170.9 ± 142.7^**^

# *t*_(9)_ = 1.88, *p* = 0.093, unpaired *t* test.

* *t*_(9)_ = 2.30, *p* = 0.047.

** *t*_(9)_ = 3.66, *p* = 0.005.

Values are mean ± SD.

All litters were born full term (G40–G41). The fraction of kits that were stillborn or died soon after birth were similar between PolyIC and PBS group (PolyIC, 46.4%, 32/69 from seven litters; PBS, 53.5%, 23/43 from four litters, χ^2^ test, χ^2^ = 0.54, *p* = 0.46). These numbers, however, were lower than in our previous study of ferret development (∼75% survival rate) that did not involve a prenatal manipulation. The body weight of the kits was higher in the PBS group than in the PolyIC group at birth but not in later stages of development (Table 3).

**Table 3.** Comparison of the body weights between the PolyIC and PBS groups for different ages

	Body weight (g)	
	PBS [*n* = 11/9 (M/F)]	PolyIC [*n* = 10/15 (M/F)]
Birth^#^	10.1 ± 0.8	8.4 ± 1.4^***^
Weaned, male	332.8 ± 20.5	322.0 ± 33.8
Weaned, Female	275.9 ± 23.2	257.6 ± 27.4
Adult^□^, male	1623.6 ± 145.2	1528.0 ± 173.9
Adult, female	796.7 ± 89.0	784.7 ± 93.0

# Sex was unspecific at birth.

□ Adults were weighted at six months old.

*** *t*_(43)_ = 3.64, *p* = 0.0007, unpaired *t* test.

Values are mean ± SD.

### Social interaction with conspecifics

**Figure 1.**
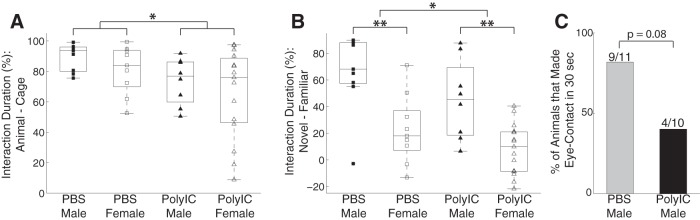
Effects of MIA on social behaviors in the ferret. ***A***, Social interaction defined as the percentage of the time interacting with a cage with another ferret minus the time interacting with an empty cage. ***B***, Social preference defined as the percentage of time interacting with a novel ferret minus the time interacting with a familiar ferret. Squares represent PBS group, and triangles represent PolyIC group; filled markers represent males and empty markers represent females. The central mark in the box plot indicates the median, and the bottom and top edges indicate 25th and 75th percentiles, respectively. ***C***, Percentage of animals that made eye-contacts with an experimenter within 30 seconds. *p < 0.05. **p < 0.01.

We performed behavioral assays once the offspring reached six months of age. Since ferrets are social animals, we first asked whether MIA changed their social behaviors by testing their interaction with other ferrets and their preference for interacting with unfamiliar versus familiar conspecifics. We found that sociability was affected by the maternal treatment conditions but not by the sex of the animals (male PBS = 89.59 ± 8.92%, *n* = 11, PolyIC = 73.51 ± 15.26%, *n* = 10; female: PBS = 81.10 ± 15.94%, *n* = 9, PolyIC = 65.55 ± 29.32%, *n* = 15; two-way ANOVA, sex, *F*_(1,41)_ = 1.48, *p* = 0.23; treatment, *F*_(1,41)_ = 5.48, *p* = 0.02; interaction, *F*_(1,41)_ = 0.00, *p* = 0.97; Fig. 1*A*). A *post hoc* multiple-comparison analysis confirmed a significant difference between the PolyIC and PBS groups (*p* = 0.02, 95% confidence interval of the difference, [2.12% 29.51%]). Given this difference, we next asked whether MIA alters the preference for engaging with familiar versus unfamiliar ferrets. We found that social preference was affected by both maternal treatment and sex (male PBS = 66.31 ± 29.45%, PolyIC = 45.12 ± 30.03%; female: PBS = 22.68 ± 25.96%, PolyIC = 8.11 ± 19.05%; two-way ANOVA, sex, *F*_(1,41)_ = 24.5, *p* = 10^−5^; treatment, *F*_(1,41)_ = 4.82, *p* = 0.03; interaction, *F*_(1,41)_ = 0.17, *p* = 0.69; Fig. 1*B*). A *post hoc* multiple-comparison analysis showed a significant difference between the PolyIC and PBS groups (*p* = 0.03, 95% confidence interval of the difference, [1.38% 34.39%]) and between males and females (*p* = 10^−5^, 95% confidence interval of the difference, [23.82% 40.32%]). Together, these findings suggest that MIA in the ferret impairs social behavior in the adult progeny.

### Social interactions with humans: eye contact

A previous study indicated that the ferret, which is a domestic species, exhibits aspects of social-cognitive skills pertaining to the interaction with humans (Hernádi et al., 2012). We adopted the method o*f* testing eye-contact tolerance from that study to examine interaction with humans. In general, the ferrets exhibited some limited periods of eye contact (cumulative duration: 0–2 s) during the 30-s test period. We characterized each animal by whether or not it made eye contact in the test period and found that animals in the PolyIC group were less likely to make eye contact with the experimenters than the ones in the control group at trend level [PolyIC = 40.0% (4/10), PBS = 81.8% (9/11), Fisher’s exact test, *p* = 0.08; Fig. 1*C*]. This result suggests that MIA impaired the social interaction with humans.

### Novel object recognition

**Figure 2.**
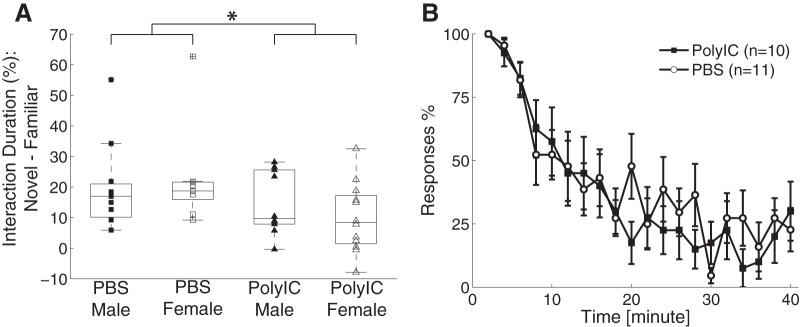
Effects of MIA on novel object recognition and adaptation to repeated auditory stimulus. ***A***, Novel object recognition defined as the percentage of the time interacting with a novel object minus the time interacting with a familiar object. Configuration the same as used in Figure 1. ***B***, Adaptation to repeated auditory stimulus. For clarity, the data are binned in 2-min windows. Error bars represent SEM. *p < 0.05.

We next probed cognitive function by investigating the ability of the ferrets to retain the memory of an object they were previously exposed to and to differentiate it from a novel object. Specifically, we tested whether the animals were able to recognize the novel object in presence of an object they were exposed to 2.5 h earlier. We characterized novel object recognition as the percentage of the time interacting with the novel object minus the percentage of time with spent with the familiar object. We found that novel object recognition was affected by the maternal treatment conditions but not by sex (male: PBS = 19.69 ± 14.01%, PolyIC = 14.52 ± 10.30%; female: PBS = 22.42 ± 15.74%, PolyIC = 9.95 ± 11.38%; two-way ANOVA, sex, *F*_(1,41)_ = 0.05, *p* = 0.82; treatment, *F*_(1,41)_ = 4.85, *p* = 0.03; interaction, *F*_(1,41)_ = 0.83, *p* = 0.37; *post hoc* multiple-comparison analysis to compare the difference between the PolyIC and PBS groups, *p* = 0.03, 95% confidence interval = [0.71% 16.92%]; Fig. 2*A*). This result suggests that MIA impairs recognition memory.

### Engagement with salient stimulus

We next asked how the ferrets responded and attended to a salient stimulus in a naturalistic setting. We attempted to engage the ferrets by rattling a noisy object in front of their home cage. The animals typically exhibited an overt redirection of their attention to the stimulus and continued to engage with it as the noisy object was moved around in front of the cage. We characterized the attention ability on a scale from 1 to 4 (for details, see Materials and Methods). We found no difference of attention level between the two groups (PBS = 2.55 ± 0.65, *n* = 11, PolyIC = 2.50 ± 0.77, *n* = 10, Student’s *t* test, *t*_(19)_ = 0.14, *p* = 0.89). The result indicates that MIA did not affect the ability to engage with and sustain attention to a salient stimulus. To expand on this finding, we next asked whether the overt attention response is sustained in response to repeat auditory stimulus application (Poole, 1972). We found that the animals attended to the source location of the stimuli for the first several presentations but gradually adapted and showed less overt attention. We characterized the response to individual auditory stimulus as attending (assigning a value of 1), partial attending (assigning a value of 0.5), or non-attending (assigning a value of 0). We found that both the PolyIC and the control group adapted to the auditory stimuli in a similar way (mean score, PolyIC = 0.38 ± 0.21, *n* = 10, PBS = 0.42 ± 0.17, *n* = 11, Student’s *t* test, *t*_(19)_ = 0.44, *p* = 0.66; Fig. 2*B*). Together, these results suggest that attentional processing is spared by MIA.

### Open field exploration

**Figure 3.**
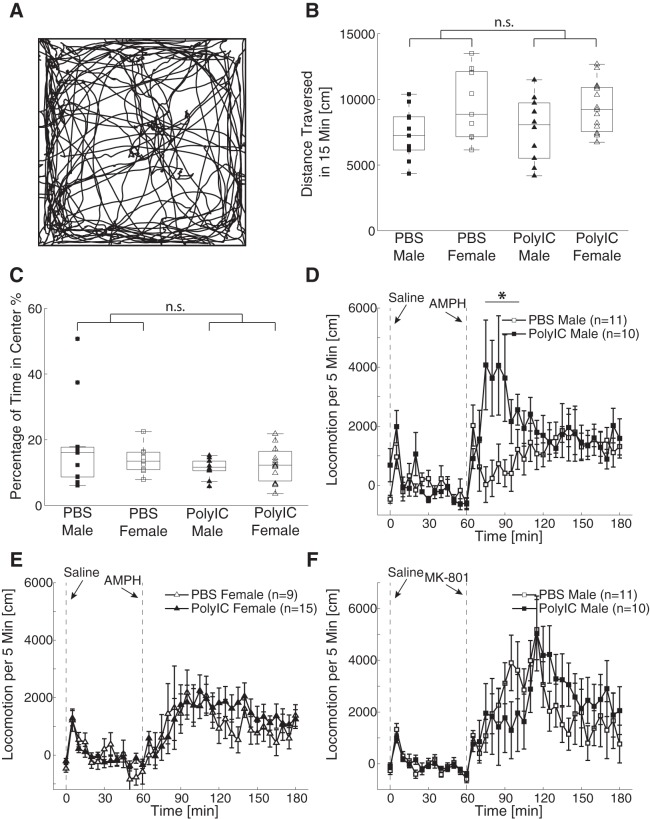
Effects of MIA on open field exploration, amphetamine- and MK-801-induced hyperlocomotion. ***A***, Example locomotion trajectory during 15 min in open field. ***B***, Distance traversed in 15 min in open field. ***C***, Percentage of time spent in the center in 15 min in open field. The configuration of ***B***, ***C*** is the same as in Figure 1. ***D***, ***E***, Time course of locomotion after amphetamine injection in males and females, respectively. ***F***, Time course of the locomotion after MK-801 injection in the male animals. Error bars indicate SEM. The vertical dashed lines at 0 and 60 min represent the time of saline and amphetamine/MK-801 injection, respectively. The locomotion activity is subtracted by baseline activity. *p < 0.05.

To control for the effects of the locomotion ability on the results observed in other behavioral tests, we investigated locomotion in an arena. We found the animals explored the arena and spent time on both the center and the periphery (Fig. 3*A*). The averaged locomotion distance was affected by the sex but not by the maternal treatment (two-way ANOVA, sex, *F*_(1,41)_ = 8.19, *p* < 0.01; treatment, *F*_(1,41)_ = 0.03, *p* = 0.86; interaction, *F*_(1,41)_ = 0.17, *p* = 0.68; Fig. 3*B*). There was no significant effect of either sex or maternal treatment on the amount of time spent in the center of the arena (two-way ANOVA, sex, *F*_(1,41)_ = 0.34, *p* = 0.56; treatment, *F*_(1,41)_ = 2.77, *p* = 0.10; interaction, *F*_(1,41)_ = 1.33, *p* = 0.26; Fig. 3*C*). Our results thus show that the results in the other behavioral assays were unlikely to be caused by changes in general locomotive patterns.

### Response to pharmacological perturbations

In rodents, MIA animals exhibit differential response in their locomotive behavior when exposed to pharmacological challenges. Therefore, we asked whether MIA ferret shared this feature with the rodent MIA models. We first studied the changes of dopamine-associated neurotransmission by testing the locomotion activity after administration of 1 mg/kg D-amphetamine. We found that the animals exhibited increased locomotive activity after the treatment. The effects of MIA on the amphetamine-induced hyperlocomotion were sex-dependent: the males in the PolyIC treatment group had more locomotion in the first hour after the amphetamine injection than the males in the PBS control group (Fig. 3*D*); however, there was no significant difference in the locomotion between the female PolyIC group and the female PBS group (Fig. 3*E*). An ANOVA analysis on the total locomotion distance within 1 h after the injection revealed a non-significant effect of sex (*F*_(1,41)_ = 1.78, *p* = 0.19), a trend-level effect of the maternal treatment (*F*_(1,41)_ = 3.39, *p* = 0.07) and a significant interaction (*F*_(1,41)_ = 4.96, *p* = 0.03). The result of the *post hoc* comparison showed that the difference of total locomotion distance in the first hour between PolyIC males and PBS control males was significant (*p* = 0.04, PolyIC = 30,409 ± 18,765 cm; PBS = 10,137 ± 14,868 cm) but the difference between PolyIC females and PBS control females was not (*p* = 0.99, PolyIC = 15,728 ± 20,937 cm, PBS = 15,399 ± 19,425 cm). Our results thus suggest the presence of sex-specific changes of dopamine-associated neurotransmission by MIA.

We next investigated changes of glutamate-associated neurotransmission by injecting the male ferrets with 0.15 mg/kg of MK-801, a non-competitive NMDA receptor antagonist. Although a visual comparison suggests that the MIA animals exhibited less locomotion in the first hour after injection and more locomotion in the second hour (Fig. 3*F*), statistical testing did not confirm this finding (first hour, PolyIC = 25,595 ± 37,229 cm, PBS = 32,488 ± 19,316 cm, unpaired *t* test, *t*_(19)_ = 0.53, *p* = 0.60; second hour, PolyIC = 27,100 ± 26,318 cm, PBS = 15,489 ± 22,183 cm, unpaired *t* test, *t*_(19)_ = 1.09, *p* = 0.29). The result suggests that the MIA-induced pharmacological changes depend on the specific type of neurotransmission.

### Gut microbiome

Given the recent finding of changes to the gut microbiome in the PolyIC mouse model (Hsiao et al., 2013), we analyzed the fecal microbiome of a subset of the animals (PolyIC/male *n* = 5, PolyIC/female *n* = 5, PBS/male = 5, PBS/female = 5) to determine whether MIA resulted in significant changes of the ferret gut microbiome. The small sample size makes this investigation a pilot study.

**Figure 4.**
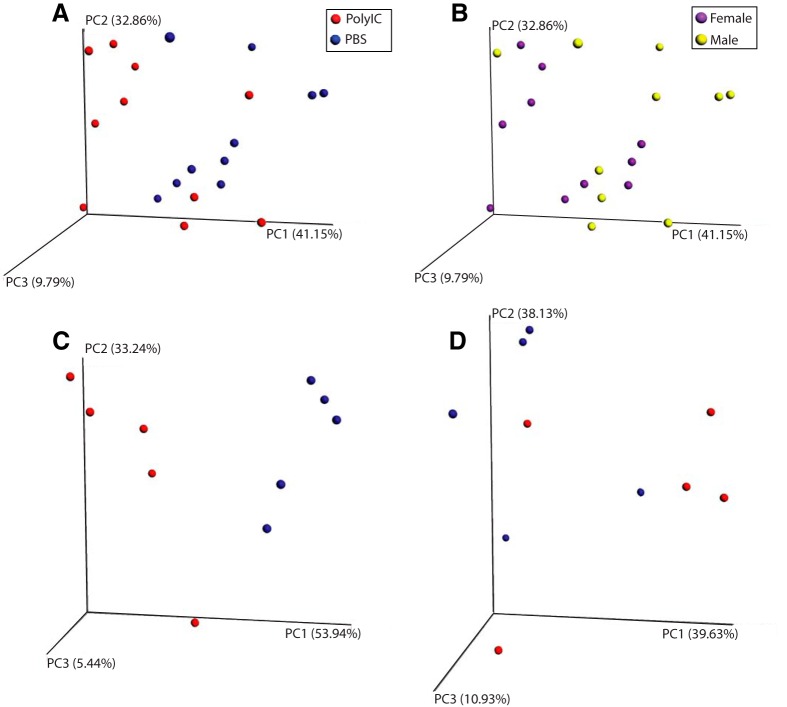
Weighted UniFrac-based PCoA plots of microbiome β diversity. Clustering of all samples (***A***, ***B***), female samples only (***C***), and male samples only (***D***). In ***A***, ***C***, ***D***, samples from PolyIC animals are colored red and samples from PBS animals are colored blue. In ***B***, female samples are colored purple and male samples colored yellow.

With weighted unifrac clustering, we found significant differences between the microbiomes of treatment and controls (*p* = 0.037; Fig. 4*A*) and between males and females (*p* = 0.029; Fig. 4*B*). When stratifying by sex, the difference between PolyIC and PBS was enhanced in females (*p* = 0.008; Fig. 4*C*) but reduced in males (*p* = 0.083; Fig. 4*D*). None of the comparisons were significant when using unweighted unifrac clustering (*p* > 0.05 for all comparisons).

We also looked for differences at the taxa level for these comparisons. One Gammaproteobacteria genus, Actinobacillus, was significantly different between treatment and controls (FDR *p* = 0.006). It was found at a low frequency in treatment samples (0.461%) but was not found in controls. There was a similar pattern for Actinobacillus in females alone (0.730% in treatment, none in controls). However, this did not reach statistical significance (FDR *p* = 0.150, uncorrected *p* = 0.005). In females alone, we found trends for differences in Clostridia (61.2% in treatment, 82.0% in controls) and two Gammaproteobacteria orders: Pasteurellales, which contains Actinobacillus (31.6% treatment, 6.91% controls) and Enterobacteriales (1.00% treatement, 9.41% controls). These differences were trending toward, but did not achieve statistical significance (FDR *p* > 0.05, uncorrected *p* < 0.05). These results are summarized in Table 4. Our results, in accordance to a similar study in mice (Hsiao et al., 2013), suggest that the changes of gut microbiome represent an important aspect of MIA.

**Table 4.** OTUs that is significant different between the PolyIC and PBS groups

	OTU	*p*	FDR_P	PolyIC	PBS
All samples	k__Bacteria;p__Proteobacteria;c__Gammaproteobacteria;o__Pasteurellales;f__Pasteurellaceae;g__Actinobacillus	0.00005	0.00624	0.46%	0.00%
Female only	k__Bacteria;p__Firmicutes;c__Clostridia	0.0472	0.44131	61.24%	82.02%
	k__Bacteria;p__Proteobacteria;c__Gammaproteobacteria;o__Pasteurellales;f__Pasteurellaceae	0.00902	0.21656	31.57%	6.92%
	k__Bacteria;p__Proteobacteria;c__Gammaproteobacteria;o__Enterobacteriales;f__Enterobacteriaceae	0.01629	0.26788	1.01%	9.41%
	k__Bacteria;p__Proteobacteria;c__Gammaproteobacteria;o__Pasteurellales;f__Pasteurellaceae;g__Actinobacillus	0.00535	0.14953	0.73%	0.00%

### Brain network dynamics in juvenile animals

**Figure 5.**
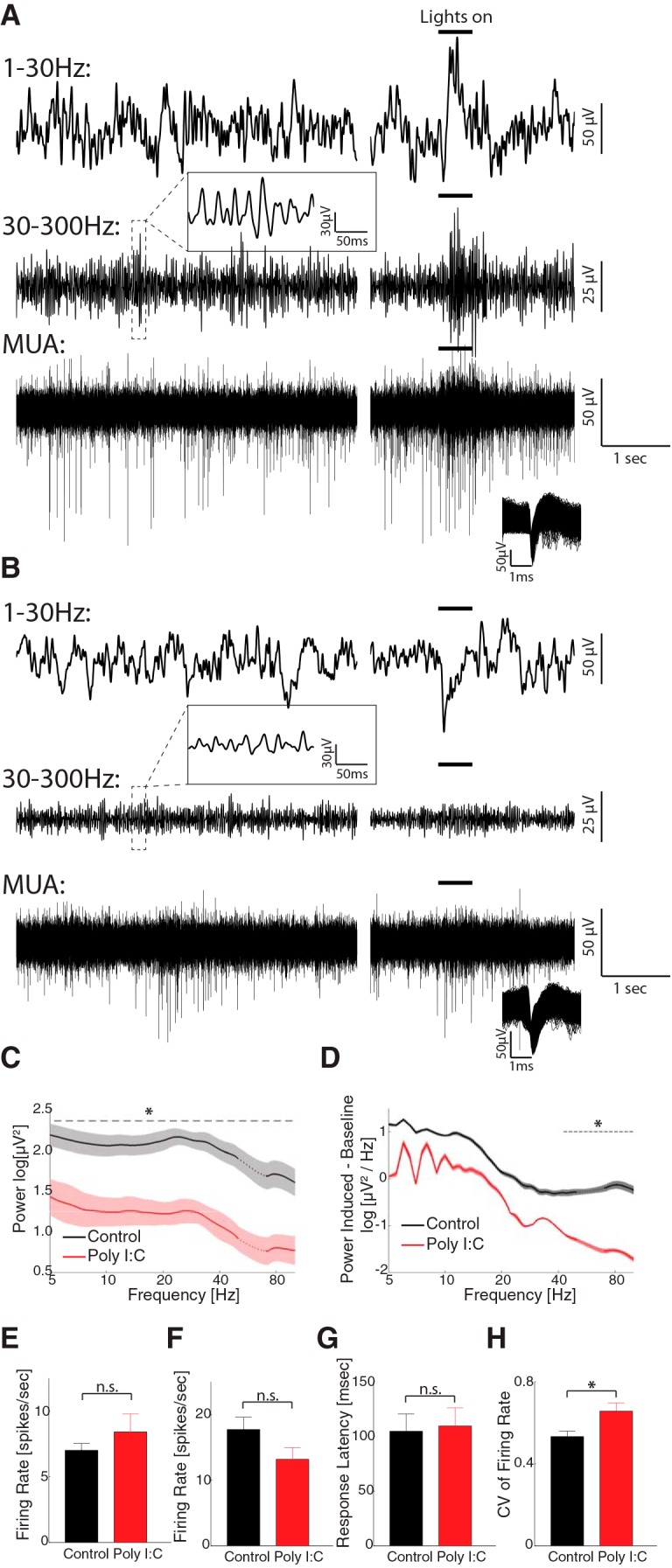
Spontaneous and visual-evoked LFP and MUAs recorded from visual cortex of MIA and control animals. ***A***, An example trace recorded from a P43 control animal. For clarity, the raw signal was band-passed filtered and shown as low-frequency LFP (1–30 Hz, top), high-frequency LFP (30–300 Hz, middle), and MUA (300–5000 Hz, bottom). The left column of plots shows the spontaneous activities. The right column displays the responses to visual stimuli whose duration is indicated by the short bold lines above. A short episode of time-resolved high-frequency LFP is shown in the inset above the trace. The inset in the bottom left of the MUA trace shows the shape of the detected spikes. ***B***, An example trace recorded from a P44 MIA animal. Same configuration as ***A***. ***C***, Power spectra (1/f normalized) of spontaneous LFP across the whole recording session in control (black, *n* = 11 animals) and PolyIC animals (red, *n* = 6 animals). Traces and shadows represent mean and SEM, respectively. The data around 60 Hz (dotted lines) are removed and interpolated between adjacent data points due to the applied notch filter. The dashed line marks the frequency range in which the power is significantly different between the two groups. ***D***, Power spectra of visually evoked activity (subtracted from the baseline power) in the control group and PolyIC group. Traces and shaded regions represent mean and SEM, respectively. The data around 60 Hz (dotted lines) are removed and interpolated between adjacent data points due to the applied notch filter. The dashed lines mark the frequency ranges in which the power is significantly different between the two periods. ***E***, Spontaneous firing rate in control group (black, *n* = 102) and PolyIC group (red, *n* = 32). Error-bar indicates SEM. ***F***, Visually elicited firing rate in control group (black, *n* = 46) and PolyIC group (red, *n* = 32). Error bar indicates SEM. ***G***, ***H***, Visually elicited response latency and CV in control group and PolyIC group, respectively. Error bar indicates SEM. n.s.: non-significant, *p < 0.05.

Previous studies have shown the MIA impairs the cortical oscillations in adult rodents with behavioral deficits (Dickerson et al., 2010, 2014; Ducharme et al., 2012). However, it is not clear whether the abnormalities in oscillations exist in juvenile animals. To answer this question, we recorded the spontaneous and visually elicited LFP and MUAs from the visual cortex in freely-moving P33–P42 animals (from three MIA litters). Six animals were recorded before eye-opening (P22–P29) and six after eye-opening (P33–P42). However, no spiking activities could be recorded from these animals before eye-opening, so we focused our analysis on data recorded after eye-opening. The data were compared to the results from control animals (Li et al., 2017). The two groups have similar age at recording (polyIC: 40.2 ± 4.5 d, range 33–45, *n* = 6, control: 39.8 ± 3.8 d, range 33–46, *n* = 8, *t*_(12)_ = 0.18, *p* = 0.86). Comparison of a representative trace recorded from a control animal (P43; Fig. 5*A*) and that from a MIA animal (P44; Fig. 5*B*) shows that while the spontaneous and visual-induced spiking activity (Fig. 5*A*,*B*, bottom traces) was generally preserved in MIA animal, the LFP amplitude were decreased, especially in the high-frequency range (top traces for 1–30 Hz and middle traces for 30–300 Hz). At the population level, maternal PolyIC administration did not significantly affect spontaneous firing rate (control, 7.02 ± 5.27 spikes/s, *n* = 102; PolyIC, 8.42 ± 7.74 spikes/s, *n* = 32, *t* test, *t*_(132)_ = 0.76, *p* = 0.45; Fig. 5*E*). The firing rate in response to the visual stimuli was decreased, yet the difference to control group was not significant (control, 17.72 ± 12.80 spikes/s, *n* = 46; after, 13.18 ± 10.12 spikes/s, *n* = 32, *t*_(76)_ = 1.67, *p* = 0.10; Fig. 5*F*). In contrast, maternal PolyIC injection significantly decreased the spontaneous LFP power throughout the frequency range we investigated (Fig. 5*C*). The visually elicited LFP power was also decreased, and the difference was significant in the high frequency-range (Fig. 5*D*). The response latency to visual stimuli was not significantly changed (control, 104.8 ± 106.7 ms, *n* = 46; PolyIC, 109.6 ± 66.4 ms, *n* = 32, *t*_(76)_ = 1.29, *p* = 0.20; Figs. 4, 5*G*). However, the CV of visual firing rate was increased in the PolyIC (control: 0.53 ± 0.18, *n* = 46; PolyIC, 0.66 ± 0.23, *n* = 32, *t*_(76)_ = 2.72, *p* = 0.008; Fig. 5*H*). Our result suggests that pathologic changes in brain oscillations may serve as a biomarker predicting the emergence of behavioral dysfunction caused by MIA.

## Discussion

Epidemiological data of neurodevelopmental disorders motivates MIA studies in animal models (Meyer and Feldon, 2010; Estes and McAllister, 2016). However, it is unclear whether the effects of MIA generalize across species with different genetic background and developmental trajectories. Furthermore, the electrophysiological properties in MIA animals, especially those in early development, remain mostly unstudied. Here, we found that MIA caused broad range of deficits/alterations in ferrets, a model species with a rich history of developmental studies, including (1) impaired sociality and social preference to conspecifics, (2) reduced social interactions with humans, (3) reduced recognition memory, (4) sex-specific increasing of amphetamine-induced hyperlocomotion, (5) altered microbiome profile, and (6) reduced high-frequency brain oscillations. Our results support MIA as an adverse factor in neurodevelopment across species.

### Alterations of behavior in adult ferrets with MIA

We found that MIA impaired social behaviors in ferrets. In agreement with our findings, previous studies showed reduced social activities/preference in MIA rodents (Shi et al., 2003; Smith et al., 2007; Bitanihirwe et al., 2010; Malkova et al., 2012). Ferrets are social animals and social behaviors are important for the development and maintenance of other behaviors (Chivers and Einon, 1982; Boyce et al., 2001). Besides affecting interactions with conspecifics, MIA also impaired interactions with humans (reduced eye contact tolerance). Since there were no group differences in our two naturalistic attention paradigms, the alterations of social behaviors are unlikely to be caused by decreased attention capabilities. It is less clear by what mechanisms MIA changes the social behavior in ferrets. One possibility is that the social behavior is changed by the alternation of the hypothalamic-pituitary-gonadal axes (Stockman et al., 1986; Vinke et al., 2008) via the action of MIA-induced cytokines (Haddad et al., 2002). Future studies are needed to test this and alternative potential mechanisms.

Our result also shows that MIA reduced the preference to novel objects. In agreement with this result, previous studies in rodents showed reduced acclimation to novel objects (Shi et al., 2003; Ozawa et al., 2006) and decreased performance in Morris water maze (Meyer et al., 2006b; Ozawa et al., 2006; Samuelsson et al., 2006; Coyle et al., 2009; Hao et al., 2010).

For the open field assay, unlike a previous study in rodent where MIA rodents spent less time in the center (Shi et al., 2003; Meyer et al., 2005), MIA ferrets spent as much time in the center as the control ferrets. The discrepancy between rodents and ferrets can be explained by the fact that ferrets are predatory animals and the time spent in center is unlikely to represent an index of “anxiety.”

We found that amphetamine-induced hyperlocomotion was increased in male MIA ferrets but not in females. MK-801 induced hyperlocomotions in male ferrets but there was no significant difference between the control and PolyIC groups. Previous studies in rodent showed enhanced amphetamine-induced hyperlocomotion (Zuckerman et al., 2003; Fortier et al., 2004; Meyer et al., 2005, 2008; Ozawa et al., 2006) and enhanced MK-801-induced hyperlocomotion (Zuckerman and Weiner, 2005; Meyer et al., 2008). Our results indicate MIA affects the pharmaco-behavior of ferret and the effects depend on the sex and specific neurotransmitter systems. Little is known about dopaminergic signaling in ferrets but previous studies showed that dopamine agonists disrupted the control of goal-directed movements, such as preying, in male ferrets (Schmidt, 1983, 1984). Our results suggest hyperactivity of the dopamine-system in ferrets by MIA, which may impact naturalistic behaviors in ferrets.

### Changes of microbiome by MIA

A previous study in mice showed that MIA also affects the gut microbiome in juvenile animals and that there is a causal link to changes in behavior (Hsiao et al., 2013). However, it is not clear whether MIA changes the gut microbiome in other species. Here, we found that MIA altered the gut microbiome in the adult ferrets. It is notable that significant changes in ferret microbiome were observed using weighted uniFrac analysis but not in the unweighted result, which suggests an altering in species richness and evenness but not the phylogenetic makeup. In contrast, Hsiao et al. (2013) found the opposite in MIA mice. The difference may come from the different species used and the ages tested. The ferrets are carnivores and receive a protein-based diet. Ferret and mouse microbiomes may have different compositions and may react differently to MIA. Furthermore, the findings from mice was from juvenile animals while our data were from adults after six months of development. Our result prompts future studies on the effects of MIA across species with different dietary habits and development time-courses. Future studies will need to investigate the relationship between the changes in gut microbiome and behavior.

Given the relatively small size of this first microbiome study in ferret, there are some important limitations to consider. In our study, the female ferrets were driving the difference between MIA and control animals. This may have to do with caging necessities: female ferrets are caged in groups where they may normalize their microbiomes to each other, while males must be single housed, likely enhancing microbiome variability across animals in either group. These differences are also more apparent when using weighted unifrac clustering, as opposed to unweighted unifrac clustering. This suggests a significant change in evenness but not in the composition of dominating species in the gut microbiome. We were able to identify some of these differences at the taxa level, which is in line with the population level differences with weighted unifrac clustering.

### Abnormal brain oscillations in juveniles

To investigate physiological changes underlying the development of the MIA phenotype, we recorded the LFP as well as the spiking activity in juvenile animals. Although the effects of MIA has been shown in behavior, anatomy, gene expression (Richetto et al., 2017), and synaptic transmission (Escobar et al., 2011; Burt et al., 2013; Patrich et al., 2016), relatively less is known about the physiologic outcome in terms of brain network dynamics. Only very few studies have focused on change of brain oscillation (Dickerson et al., 2010, 2014; Ducharme et al., 2012). Our result of LFP and MUA recordings show that, while the firing rate was not significantly changed, a reduction of spontaneous and sensory-evoked neural oscillations occurred in this early developmental stage. This suggests that the impairment of neural synchronization, which is to some extent independent of individual neuronal firing, may be a prominent phenomenon before the appearance of many behavioral phenotypes. Further studies will need to investigate the potential to use the neural oscillation and synchronization as a biomarker to predict psychiatric disorders or guide prevention and treatment. The diagnostic potential of this finding is supported by a recent study in which applying deep brain stimulation to medial prefrontal cortex in adolescence prevented the behavioral deficits and anatomic abnormalities associated with MIA in adult rats (Hadar et al., 2018).

### Limitations

As any scientific study, our work has a series of limitations. First, we did not cross-foster the kits on birth to control for the nurture effect. Previous cross-foster study in rodents showed that both prenatal insult and postnatal adoption by PolyIC-treated mothers will impair the behavior of rat offspring in adulthood (Meyer et al., 2006a). Future experiments will need to include cross-fostering of the ferret kits. Second, no systematic dosing study of PolyIC was done, nor did we parameterize the timepoint of the PolyIC injection during gestation. This choice was the result of cost considerations. Previous studies showed dose-dependent effects of prenatal PolyIC injections (Shi et al., 2003; Meyer et al., 2005). In the present study, we used a dosage and administration route similar to previous rodent studies (Shi et al., 2003; Smith et al., 2007). The result of similar offspring survival rates for the MIA and control groups suggests that the chosen dose is safe. The exact effects of prenatal PolyIC also depend on the gestational stage at the injection (Meyer et al., 2006b). Here, the insult age was in mid-late gestation stage and corresponds to the second trimester in human (Clancy et al., 2007). We chose this time point as a critical time point in genesis of cortical neurons (Jackson et al., 1989) and formation of the thalamocortical connections (Johnson and Casagrande, 1993) in ferrets. Future studies are required to test the effect of MIA at other gestational ages. Third, due to the limited litter size, data from all animals from all litters were pooled together in the data analysis, which means that the result could be biased because of the uneven litter size and within-litter effects. Fourth, we used the data set of electrophysiological recording from a previous study as control when comparing the results of MIA animals. Thus, we cannot exclude that some of the differences are a consequence of the additional procedures that were performed on both groups in the present study but not in our previous study on brain development in the healthy ferret. We thus emphasize that these results are to be considered preliminary and exploratory. Fifth, in the study all male ferrets were single-housed when they grew up and became progressively aggressive. Sixth, the shipment during pregnancy might induce stress and potentially affected the development, although we chose the safest gestation period as advised by the animal supplier. Seventh, we did not perform a behavioral characterization during development to exclude that testing procedures affected brain maturation and development. Eighth, the gut microbiome study was underpowered and are thus also preliminary and exploratory.

In summary, we tested whether MIA alters behaviors, brain oscillations, and the gut microbiome in ferrets, a predator which is distinct to the laboratory rodents both in evolution and behaviors. Indeed, we found changes in behaviors similar to the phenotypes in MIA models of other species, supporting that the detrimental effects of MIA in neurodevelopment are universal across species. Our results suggest the possibility to model neurodevelopmental disorders in ferrets. Furthermore, the findings of the alternations of gut microbiome in adults and the decrease of higher frequencies oscillation power in juveniles demonstrate the feasibility to use this model to test hypotheses about the biological mechanisms underlying the environmentally induced developmental perturbations.

## Synthesis

Reviewing Editor: Cheryl Sisk, Michigan State University Decisions are customarily a result of the Reviewing Editor and the peer reviewers coming together and discussing their recommendations until a consensus is reached. When revisions are invited, a fact-based synthesis statement explaining their decision and outlining what is needed to prepare a revision will be listed below. Note: If this manuscript was transferred from JNeurosci and a decision was made to accept the manuscript without peer review, a brief statement to this effect will instead be what is listed below.

Please revise your manuscript to address the following points. In addition, please carefully proof-read the manuscript for typographical and grammatical errors.

Line 113: should read ‘used in rodents’

Lines 130-32: text is not the same as stated in response to reviewers

Line 155: should read ‘any of these treatments’

Line 165: should read ‘open field’

Lines 190-92: Still needs rewording. Suggest “locations of the cage that contained the familiar animal or the stranger animal”, or “locations of the cages containing the familiar animal and the stranger animal”.

Line 372: should read ‘involve’

Lines 376-93: Authors state in response to reviewers that they added this sentence to this section: “The fact that males had higher sociability and social preference may be because they were single-housed.” I cannot find this sentence.

Line 543: this sentence needs to be re-worded: “...result suggests use MIA ferrets to model...”

Line 608: should read ‘medial prefrontal cortex’

Line 611, Limitations section: Text should be added to acknowledge that the electrophysiology and microbiome experiments should be considered preliminary and exploratory. Reviewers challenged the authors on these experiments, which either lacked proper controls or were underpowered. Authors agree with these points in their response to reviewers but do not explicitly or sufficiently identify these studies as preliminary or exploratory.
